# Differences in the Fatty Acid Profile, Morphology, and Tetraacetylphytosphingosine-Forming Capability Between Wild-Type and Mutant *Wickerhamomyces ciferrii*

**DOI:** 10.3389/fbioe.2021.662979

**Published:** 2021-06-09

**Authors:** Jun Young Choi, Hee Jin Hwang, Woo Yeon Cho, Jong-il Choi, Pyung Cheon Lee

**Affiliations:** ^1^Department of Molecular Science and Technology, Ajou University, Suwon, South Korea; ^2^Department of Applied Chemistry and Biological Engineering, Ajou University, Suwon, South Korea; ^3^Department of Biotechnology and Bioengineering, Chonnam National University, Gwangju, South Korea

**Keywords:** *Wickerhamomyces ciferrii*, tetraacetylphytosphingosine, sphingolipid, fatty acid, mutation

## Abstract

One tetraacetylphytosphingosine (TAPS)-producing *Wickerhamomyces ciferrii* mutant was obtained by exposing wild-type *W. ciferrii* to γ-ray irradiation. The mutant named 736 produced up to 9.1 g/L of TAPS (218.7 mg-TAPS/g-DCW) during batch fermentation in comparison with 1.7 g/L of TAPS (52.2 mg-TAPS/g-DCW) for the wild type. The highest production, 17.7 g/L of TAPS (259.6 mg-TAPS/g-DCW), was obtained during fed-batch fermentation by mutant 736. Fatty acid (FA) analysis revealed an altered cellular FA profile of mutant 736: decrease in C16:0 and C16:1 FA levels, and increase in C18:1 and C18:2 FA levels. Although a significant change in the cellular FA profile was observed, scanning electron micrographs showed that morphology of wild-type and mutant 736 cells was similar. Genetic alteration analysis of eight TAPS biosynthesis-related genes revealed that there are no mutations in these genes in mutant 736; however, mRNA expression analysis indicated 30% higher mRNA expression of *TCS10* among the eight genes in mutant 736 than that in the wild-type. Collectively, these results imply that the enhancement of TAPS biosynthesis in mutant 736 may be a consequence of system-level genetic and physiological alterations of a complicated metabolic network. Reverse metabolic engineering based on system-level omics analysis of mutant 736 can make the mutant more suitable for commercial production of TAPS.

## Introduction

Sphingolipids are a class of lipids containing a long-chain base (LCB) backbone (known as a sphingoid base) and are essential components of eukaryotic cellular membranes (Gault et al., 2010; Börgel et al., 2012; Singh and Del Poeta, 2016). The simple structures of LCBs, including those of sphingosine, dihydrosphingosine, and phytosphingosine, form diverse sphingolipids via modifications such as phosphorylation or acylation. Aside from their key role as a component of cellular membranes, sphingolipids perform other important functions in eukaryotes, including signal transduction, the heat stress response, acid/alkaline tolerance, endocytosis, and cell division (Jenkins et al., 1997; Menaldino et al., 2003; Rittershaus et al., 2006; Cowart and Obeid, 2007; Epstein et al., 2012; Montefusco et al., 2014; Singh and Del Poeta, 2016).

**FIGURE 1:**
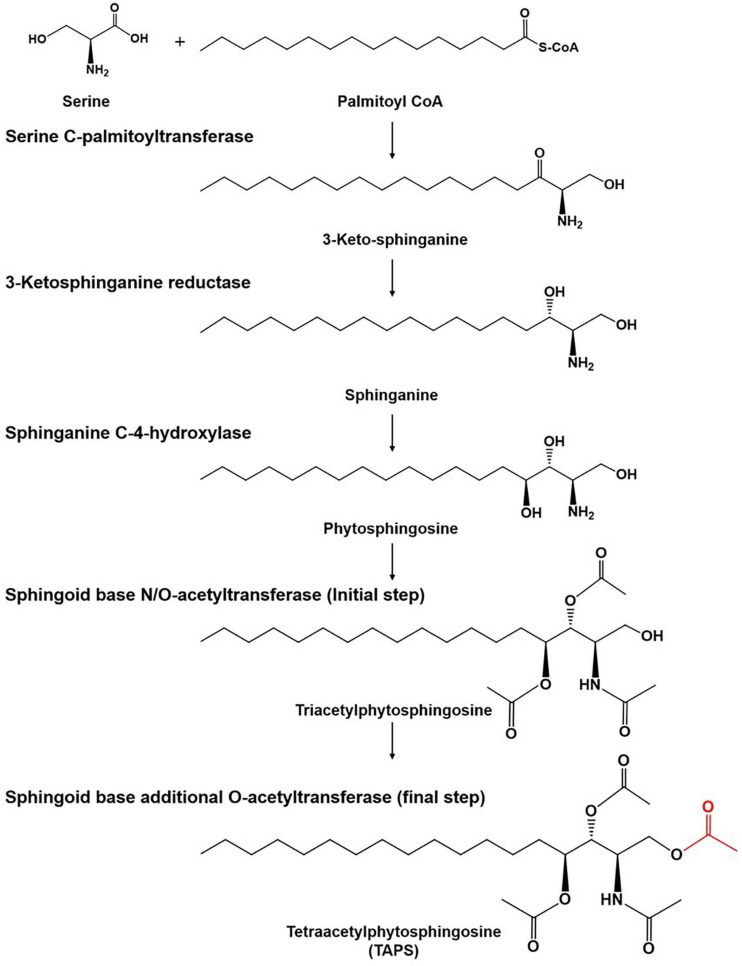
The tetraacetlyphytosphingosine (TAPS) biosynthetic pathway in *W. ciferrii.* The serine-palmitoyltransferase (SPT) complex composed of Lcb1, Lcb2, and Tsc3 catalyzes the first condensation reaction, between serine and palmitoyl-CoA, to produce 3-keto-sphinganine. The next step is mediated by 3-keto-sphinganine reductase (Tsc10) and yields sphinganine. Sphinganine C-4-hydroxylase (Syr2) adds a hydroxyl group to sphinganine to produce phytosphingosine. Sphingoid base N-/O-position acetyltransferase (Sli1p) and O-position acetyltransferase (Atf2p) are sequentially involved in acetylation reactions and produce TAPS (the final acetylated moiety is highlighted in red) as a final product. Three compounds (triacetyl sphinganine, sphinganine-1-P, and phytosphingosine-1-P) are destined for other metabolic pathways.

There is growing interest in the use of sphingolipids and their derivatives, such as ceramides, as cosmetic ingredients as they are one of the most important components in the human skin and have high moisture retention capacity (Coderch et al., 2003; Kim et al., 2014; Murakami et al., 2015). Sphingolipids are usually obtained by chemical extraction from natural sources as well as via *de novo* enzymatic reactions (Bae et al., 2003; Zhang et al., 2006; Reisberg et al., 2017). As an alternative producer of sphingolipids, the budding yeast *Wickerhamomyces ciferrii* (reclassified and renamed *Pichia ciferrii*) has been recognized as a microbial production host for sphingolipids, including tetraacetylphytosphingosine (TAPS). Notably, *W. ciferrii* naturally secretes a considerable amount of TAPS, thus, facilitating TAPS purification in a downstream process (Barenholz and Gatt, 1972). The fermentation-derived TAPS is efficiently converted by deacetylation into phytosphingosine, which is further modified into ceramides by ceramide synthase with fatty acyl-coenzyme A (CoA) as a cosubstrate (Wells et al., 1998; Schneider et al., 2012; Kim et al., 2014). The TAPS biosynthetic pathway in *W. ciferrii* is known to involve four enzymatic reactions, starting from a reaction between serine and palmitoyl-CoA (Figure 1). The rate-determining reaction step is the condensation of serine and palmitoyl-CoA by heterodimeric serine C-palmitoyltransferase (encoded by genes *LCB1* and *LCB2*), resulting in 3-keto-sphinganine. The latter is then reduced to sphinganine by 3-keto-sphinganine reductase (encoded by the *TSC10* gene). Sphinganine is hydroxylated into phytosphingosine by sphinganine C-4-hydroxylase (encoded by the *SYR2* gene). Last, phytosphingosine is acetylated, meaning that TAPS is formed at this point by sphingoid base N/O-acetyltransferases (encoded by the genes *Slli1* and *Atf2*) (Börgel et al., 2012; Schorsch et al., 2012; Ter Veld et al., 2013).

To date, *W. ciferrii* has been exclusively used as a microbial strain for TAPS production. Several efforts including metabolic engineering have been made to increase the titer of TAPS generated by *W. ciferrii*. The current reported titer of TAPS ranges from approximately 0.2 to over 2 g/L when produced by genetically engineered *W. ciferrii* grown with glucose as a carbon source (Börgel et al., 2012; Schorsch et al., 2012). A recent study reported that a genetically engineered *Yarrowia lipolytica* could produce up to 0.65 g/L TAPS in fed-batch cultures with glycerol as a carbon source (Han et al., 2020). Notably, glycerol is increasingly employed as a renewable low-cost carbon source in yeast fermentations for lipid production (Papanikolaou et al., 2017a; Souza et al., 2017). Utilization of crude glycerol as a carbon source for growing *W. ciferrii* would be one of cost-effective strategies for the commercial scale TAPS production.

On the other hand, a draft genome sequence (15.9 Mb, 364 contigs) of wild-type *W. ciferrii* may point to additional target gene(s) or regulatory site(s) involved in TAPS biosynthesis (Schneider et al., 2012). Although the rational genetic/metabolic engineering of several targets within the genome has significantly enhanced TAPS production, there are still unknown target genes that are difficult to identify with rational approaches. Mutant breeding has been one of the alternative methods for permanently altering a gene(s) to obtain desired features (Tale et al., 2018). This technique usually requires physical mutagens, such as ultraviolet (UV) light and γ-rays (Lemontt, 1971; Lawrence et al., 1985; Sitepu et al., 2012) to induce mutations within a target genome, and then the mutant strains must be screened to identify those with the desired features.

In this study, TAPS-producing wild-type *W. ciferrii* was physically mutagenized by γ-ray irradiation to obtain mutants with enhanced TAPS production. A mutant named 736, which overproduces TAPS, was selected and systematically compared with the wild-type strain.

## Materials and Methods

### Strains, a Mutant Library, and Screening of the Mutants

Wild-type *W. ciferrii* (strain F-60-10A NRRL1031) served as the parental strain for our mutation study. Wild-type and mutant *W. ciferrii* strains were aerobically grown in the YMgI medium (0.7% of bactopeptone, 0.2% of malt extract, 0.2% of yeast extract, and 3% of glycerol) supplemented with 10 mM CaCl_2_ and 5 g/L serine (designated as the YMgISC medium), if necessary. Cell growth was monitored at a wavelength of 600 nm (OD_600_) with a SPECTRAmax PLUS384 spectrophotometer (Molecular Devices, United States). A 1.5 ml microtube containing 1 ml of wild-type *W. ciferrii* culture (OD_600_ = 1.0) was γ-irradiated at 0.3 kGy in ^60^Co γ-irradiator IR-79 (Nordion International Ltd., Ontario, Canada). After that, the cells were immediately spread on YMgISC agar plates and incubated at 25°C until colonies formed on the agar plates (∼4 days). The colonies were then inoculated into 0.2 ml of the YMgISC medium in each well of 96-deep-well plates and were incubated at 25°C for 3 days. A Nile red (Sigma-Aldrich, United States) solution (2 μg of Nile red per milliliter of dimethyl sulfoxide) was added in equal amounts to 50 μl of the culture medium to stain TAPS of the cells. Fluorescence intensity of the stained cells was measured on a SPECTRAmax Gemini plate reader (Molecular Devices) with excitation at 530 nm and emission at 590 nm (Jarvis et al., 2004; Choi et al., 2014).

### Sequencing of TAPS Pathway Genes in the Wild-Type Strain and Mutant 736

Genomic DNA samples were extracted from cells of the wild-type and mutant 736 strains, which were aerobically grown in the YMgI medium, using a Genomic DNA Kit (Macrogen, South Korea). Eight genes (*LCB1*, *LCB2*, *LCB3*, *TSC3*, *TSC10*, *SYR2*, *SLI1*, and *ORM12*) of the wild-type strain and mutant 736 were sequenced with gene-specific primers (Table 1) using the Sanger method. Gene-specific primer sequences were designed based on the published draft genome sequence data from wild-type *W*. *ciferrii*^1^ (Schneider et al., 2012).

**TABLE 1:** Primers used in this study.

**Gene**	**Sequence (5′–3′)**
*LCB1*	F: AGTAATGGTGTTGGTGCTTGTG/R: TCAGCAATAACAAAATCAC CTCTTT
*LCB2*	F: GGTCAACCAAGATCTCATCGTC/R: AACACCACGACCTGATGGAC
*LCB3*	F: TGTGGATATGGACGTGAAGCA/R: CCGCTGTAGCATTTGCACTA
*TSC3*	F: ACGAAGAGCGTGACAAGGATT/R: TGGTAAATAGGCAAAAA CGGCAT
*TSC10*	F: ACTGCAGTTAATTTTGCTCATGC/R: AGCTTCTTGTCTCAG GACATCA
*SYR2*	F: ACCTTAGGTACCGGTATTGCTG/R: TGGTCTTGATACCAAAT TGTTGATG
*SLI1*	F: TGGTGCATATGATGATTGGGG/R: ATCAACAGCTGACTTTCCCA
*Orm12*	F: ACAACACATGAACCAATTTCTGTTG/R: AAGCACCTTTTGAAT GAACCCA

### Reverse-Transcription (RT) PCR

For the RT-PCR analysis of TAPS pathway genes of the wild-type and mutant 736 strains, total RNA was extracted from the cells in the mid-exponential and stationary growth phases using the Hybrid-R RNA Purification Kit (GeneAll Biotechnology, South Korea). cDNA synthesis from the total-RNA samples was carried out with the ReverTra Ace qPCR-RT Kit (Toyobo, Japan). RT-PCR analysis of eight genes (*LCB1*, *LCB2*, *LCB3*, *TSC3*, *TSC10*, *SYR2*, *SLI1*, and *ORM12*) was conducted on a Rotor gene Q Real Time PCR machine (Qiagen, Germany). Gene-specific primers (Table 1) were designed based on the genome sequence data from wild-type strains. The *pfk* gene, encoding phosphofructokinase, served as the reference gene for RT-PCR.

### Scanning Electron and Light Microscopy

Morphological changes in colonies of the wild-type strain and mutant 736 were periodically analyzed with light microscopy (Laborlux K; Leitz, Germany): the cells were grown on agar plates for 192 h before this analysis. Scanning electron micrographs of the wild-type and mutant *W. ciferrii* strains were obtained by means of a JSM 5410LV scanning electron microscope (JEOL, Japan), as described elsewhere (Lawton, 1989).

### Flask and Bioreactor Fermentation Procedures

For flask fermentation, a single colony of *W. ciferrii* from a YMgI agar plate was incubated in a 15-ml glass culture tube with 3 ml of the YMgI medium (2 g/L of yeast extract, 2 g/L of malt extract, and 7 g/L of peptone), containing 10 g/L of glycerol, at 25°C with shaking at 180 rpm. When precultured cells reached an OD_600_ of approximately 5, 3 ml of the preculture was inoculated into a 500 ml baffled flask containing 50 ml of YMgI, YMgIS (YMgI + 5 g/L of serine), YMgIC (YMgI + 10 mM CaCl_2_), or YMgISC (YMgI + 5 g/L of serine + 10 mM CaCl_2_) supplemented with 10 g/L of glycerol and then grown at 25°C with shaking at 180 rpm for 48 h. For batch bioreactor fermentation, 100 ml of the preculture cell suspension was incubated in a 3-L BIOSTAT B jar bioreactor (Sartorius, Germany) containing 1.5 L of the YMgISC medium supplemented with either 35 or 130 g/L glycerol. The dissolved oxygen (DO) level was maintained at 30% by supplying either air or a mixture of air and pure oxygen gas. The temperature was maintained at 25°C, and pH was automatically maintained at 5.6 by the addition of a 5N NH_4_OH solution. Two concentrations of glycerol (35 and 130 g/L) were used as an initial carbon source in batch fermentation.

For fed-batch fermentation, 100 ml of the preculture cell suspension was incubated in the 3-L BIOSTAT B jar bioreactor containing 1.5 L of the YMgISC medium supplemented with 50 g/L of glycerol. The DO level was maintained by increasing the agitation rate from 300 to 1,000 rpm and by supplying air and pure O_2_ gas at 1.5 vvm (vol/vol/min). pH and temperature were controlled with the method described for the batch fermentation above. When the initial glycerol was depleted, a feeding solution (230 g/L glycerol) was periodically added to maintain a residual glycerol concentration of 0–10 g/L in the media via the DO-stat feeding method. Cell growth was monitored by the measurement of OD_600_ on a SPECTRAmax Plus384 spectrophotometer (Molecular Devices, United States). Dry cell weight (DCW) was calculated using the linear relationship (*R*^2^ = 0.999) between OD_600_ and DCW: 1.0 of OD_600_ is equivalent to 0.489 mg-DCW/L. The concentration of glycerol was determined using an Agilent 1100 HPLC system equipped with a refractive index detector (Agilent 1100) and an Aminex HPX-87H column (7.8 × 300 mm, Bio-Rad, United States) at a flow rate of 0.7 ml/min with 4 mM H_2_SO_4_ as a mobile phase.

### Extraction and Analysis of TAPS and Cellular Fatty Acids

For TAPS analysis, 1 ml of culture was harvested, and the collected cells were extracted twice with 1 ml of methanol. Hexadecane (200 mg/L) was added as an internal standard for the quantification of TAPS. The collected methanol extracts were incubated with 0.1 g of sodium sulfate at 25°C for 30 min and then centrifuged at 12,000 rpm for 30 min. After that, the supernatants were evaporated in an EZ-2 dryer (Genevac, United Kingdom), and the dried samples were dissolved in 1 ml of methanol and analyzed with gas chromatography coupled with mass spectrometry (GC-MS) on a system (models 7890 and 5975; Agilent Technologies, United States) equipped with an HP-5MS capillary column (30 m × 250 μm × 0.25 μm, Agilent Technologies). Commercial TAPS was purchased from Sigma-Aldrich and was employed as the standard for quantification. For fatty-acid analysis, a cell suspension with an OD_600_ of approximately 25, grown in a bioreactor, was subjected to fatty-acid extraction using the method described previously (Diaz et al., 2008). Fatty-acid methyl esters were analyzed by means of the GC-MS system (models 7890 and 5975) equipped with an HP-5MS capillary column, with the help of a fatty-acid methyl ester standard (Sigma Aldrich) and the NIST mass spectral database.

### Statistical Analysis

Results are expressed as mean ± standard deviation of technical replicates (*n* = 3 for fermentation, and *n* = 10 for fatty acid analysis). *P*-values of <0.05, <0.01, or <0.001 were used to denote significant differences between mean values, as determined with one-way analysis of variance (ANOVA) performed in SigmaPlot 12.0 (Systat Software Inc., San Jose, CA, United States).

## Results

### Screening of TAPS-Overproducing Mutant *W. ciferrii* Strains

To obtain such strains, a γ-irradiation technique (at a dose of 0.3 kGy) was applied to the wild-type *W. ciferrii* strain, as previously reported (Lawrence et al., 1985; Jarvis et al., 2004). In a clone library of 10^5^ colony-forming unit (CFU) size, approximately 3,000 clones showing a growth rate similar to that of the wild-type strain on an agar plate were cultured in 96-deep-well plates. As no specific stain/dye that binds to TAPS is available, a Nile red–based screen was utilized as an initial screening method, even though the false positive rate was ∼ 40%. Although Nile red staining has been frequently used for microbial strains overproducing an intracellular lipid (Choi et al., 2014; Rumin et al., 2015), we confirmed that Nile red staining of purified TAPS and TAPS-producing cells was TAPS-concentration dependent. After incubation at 25°C for 3 days, cell cultures in 96-well plates were stained with Nile red and then initially screened based on their fluorescence intensity. Clones showing high fluorescence intensity were cultured in a 15 ml flask, and TAPS titers were determined. Among the clones screened, a mutant named 736, producing a higher TAPS titer than the wild-type strain, was chosen and further characterized.

### Morphological Characterization of Mutant 736 and the Wild-Type Strain

**FIGURE 2:**
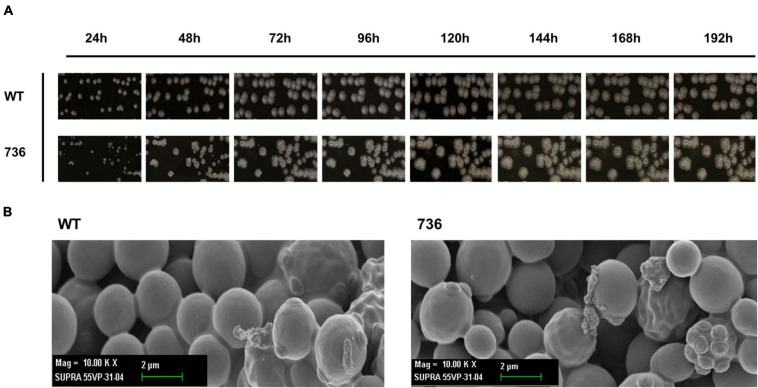
Morphological changes in the wild-type and mutant 736 on agar plates and scanning electron micrographs. **(A)** Morphological changes in the wild type (indicated as WT) and mutant 736 (indicated as 736) on YPD agar plates were periodically compared for 192 h. **(B)** A scanning electron micrograph of the wild-type (left) and mutant 736 (right).

First, we compared the morphological changes between colonies of the mutant 736 strain and wild-type colonies on agar plates. As previously reported (Wickerham and Stodola, 1960), colonies of the wild-type strain changed gradually to slimed and mildly wrinkled morphology at 48 h (Figure 2A). In contrast to the morphological characteristics of the wild-type colonies, severely wrinkled colonies with crystal-like substances developed at 48 h on the agar plates where mutant 736 was growing (Figure 2A). The severely slimed and irregular crystal-like substances around the colonies of mutant 736 continued to increase in amount at 192 h. Scanning electron microscopy revealed that there were no distinguishable differences in the morphological characteristics between the wild-type and mutant 736 strains (Figure 2B). Therefore, the irregular crystal-like substances around the colonies of mutant 736 strains could be aggregated forms of secreted TAPS, suggesting higher formation of TAPS in mutant 736 than in the wild-type strain.

### Characterization of Fatty Acid Profile of the Mutant 736 Strain and Wild Type

**FIGURE 3:**
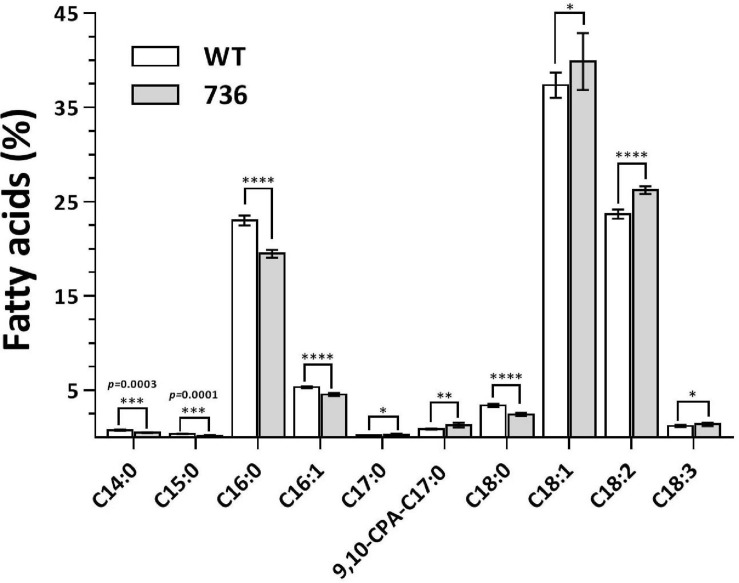
Fatty-acid composition of the wild-type and mutant 736. Fatty acids of the wild-type (blank) and mutant 736 (gray) were extracted and analyzed with GC-MS. Statistical analysis was performed using one-way ANOVA (^∗^*p* < 0.05, ^∗∗^*p* < 0.01, ^∗∗∗^*p* < 0.001, ^****^p < 0.0001, *n* = 10). Data are presented as mean ± SD.

We determined the fatty-acid composition of the wild type and mutant 736 with GC-MS (Figure 3 and Supplementary Figure 1) because a fatty-acid composition change could explain the TAPS-overproducing capability of mutant 736. The major fatty acid (>4% of the total amount of fatty acids) of the wild type was C18:1 (37.4%), followed by C18:2 (23.7%), C16:0 (23.0%), and C16:1 (5.3%), which were in a good agreement with the previous study (Papanikolaou et al., 2017b). Similarly, the major fatty acid of mutant 736 was C18:1 (39.9%) followed by C18:2 (26.2%), C16:0 (19.5%), and C16:1 (4.6%). The minor fatty acids (<4% of the total amount of fatty acids) of the wild type and mutant 736 were C14:0, C15:0, C17:0, 9, 10-CPA-C17:0, C18:0, and C18:3 (Figure 3 and Supplementary Table 1). Notably, the observed decreased percentage of C16:0 (from 23.0 to 19.5%) and C16:1 (from 5.3 to 4.6%) and increased percentage of C18:1 (from 37.4 to 39.9%) and C18:2 (from 23.7 to 26.2%) in mutant 736 might be indirectly related to enhanced TAPS formation in mutant 736 because fatty acid C16:0 represents one of two precursors (C16:0 palmitoyl-CoA and serine) in the biosynthesis of TAPS (Figure 1). We can speculate that mutant 736 increased C18:1 and C18:2 production to physiologically adapt to the shortage of C16:0 and C16:1, which are involved in the maintenance of membrane function and fluidity.

### mRNA Expression of Eight TAPS Biosynthesis-Related Proteins in the Mutant 736 Strain and Wild-Type

**FIGURE 4:**
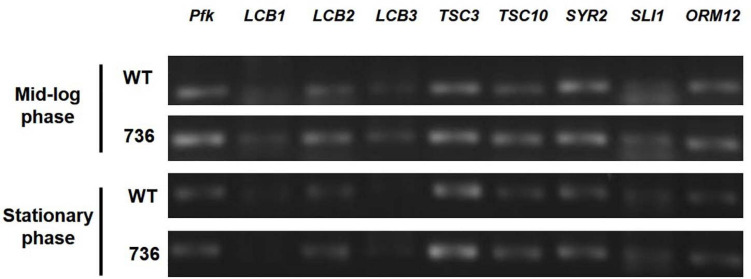
Transcriptional analysis of eight TAPS biosynthesis–related genes in mutant 736 and in the wild-type in mid-log and stationary phases. RT-PCR analysis of eight genes (*LCB1*, *LCB2*, *LCB3*, *TSC3*, *TSC10*, *SYR2*, *SLI1*, and *ORM12*) extracted from the mutant 736 (indicated as 736) and wild-type (indicated as WT) cells in mid-log and stationary growth phases. Housekeeping gene *pfk* served as the reference.

Next, we hypothesized that expression alterations of TAPS biosynthesis-related proteins are responsible for the observed higher TAPS-forming capability of mutant 736. Therefore, we compared transcriptional levels of the eight TAPS biosynthesis-related genes—*LCB1*, *LCB2*, *LCB3*, *TSC3*, *TSC10*, *SYR2*, *SLI1*, and *ORM12*—between mutant 736 and wild-type cells grown to mid-log and stationary growth phases (Figure 1). As presented in Figure 4, no dramatic changes (>twofold change) in mRNA levels of the eight genes between the wild type and mutant 736 were observed in either mid-log or stationary-phase cells. Notably, only *TSC10* was expressed at a higher level (by ∼30%) in mutant 736 than in the wild type in both mid-log and stationary growth phases. This overexpression could explain why mutation-induced upregulated expression of the TAPS pathway gene might be one of the factors behind the observed higher TAPS titers in mutant 736. Next, we investigated genetic variation of the eight TAPS biosynthesis–related proteins of both the wild type and mutant 736, using Sanger sequencing. Unexpectedly, the eight gene sequences were identical between mutant 736 and the wild-type (Supplementary Figure 2), indicating no changes in intrinsic enzymatic activity or other properties. Although we did not confirm the protein expression levels of the eight enzymes, we strongly believe that systems level mutations in the genome of mutant 736 could be one of the factors explaining enhanced TAPS production in mutant 736, on the basis of the observed differences in phenotypes and TAPS-producing capability between the wild-type and mutant 736.

### TAPS Production by the Mutant 736 Strain and Wild-Type During Flask Cultivation

Mutant 736 and the wild-type were cultivated in baffled flasks containing one of four media (YMgI, YMgIS, YMgIC, or YMgISC) supplemented with 10 g/L glycerol, to investigate the effects of the addition of serine and/or CaCl_2_ on TAPS production. Among the four media, YMgISC yielded the highest TAPS production in mutant 736 (892 ± 97 mg/L) and wild-type (442 ± 27 mg/L), followed by YMgIC (781 ± 96 mg/L in mutant 736 vs. 420 ± 39 mg/L in the wild-type), YMgIS (447 ± 11 mg/L in mutant 736 vs. 327 ± 35 mg/L in the wild-type), and YMgI (404 ± 70 mg/L in mutant 736 vs. 282 ± 71 mg/L in the wild type). Notably, the TAPS titer in mutant 736 was twice as high as that of the wild type (892 ± 97 vs. 442 ± 27 mg/L in YMgISC). The higher TAPS titers in mutant 736 and wild-type in the YMgIS, YMgIC, and YMgISC media in comparison with the YMgI medium suggested that the addition of serine and CaCl_2_ had a positive influence on TAPS production in both mutant and wild-type strains.

### TAPS Production by the Mutant 736 Strain and Wild Type During Bioreactor Cultivation

**FIGURE 5:**
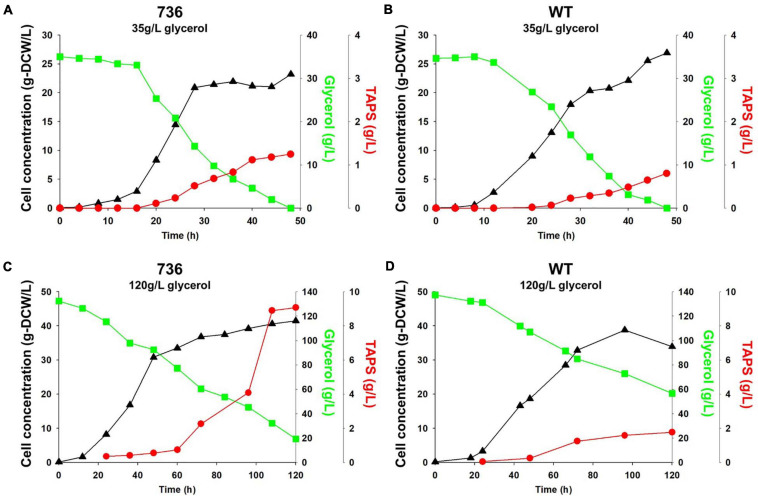
Growth and TAPS titers of mutant 736 and the wild-type in batch fermentation procedures with 35 and 130 g/L of glycerol as a carbon source. Mutant 736 **(A)** and the wild-type **(B)** in the YMgISC medium supplemented with 35 g/L of glycerol as a carbon source. Mutant 736 **(C)** and the wild-type **(D)** in the YMgISC medium supplemented with 130 g/L of glycerol as a carbon source. Symbol Δ represents cell concentration; □, glycerol; •, TAPS.

**FIGURE 6:**
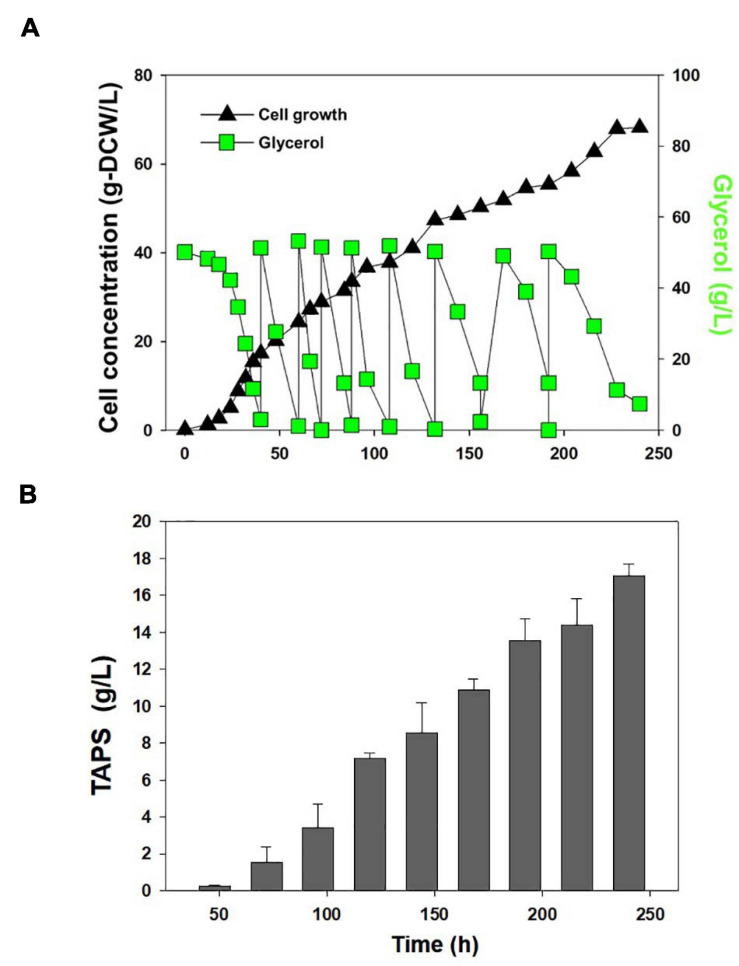
Fed-batch fermentation of mutant 736. **(A)** Cell growth and glycerol consumption kinetics of mutant 736 during fed-batch culture. A triangle represents cell concentration; a square, glycerol. **(B)** TAPS concentration during fed-batch culture.

Cell growth and TAPS production of the mutant 736 and wild-type strain were investigated with batch bioreactor fermentation at two initial glycerol concentrations (35 and 130 g/L). When cultured in the YMgISC medium containing 35 g/L of glycerol, mutant 736 produced up to 1.2 g/L of TAPS (53.8 mg-TAPS/g-DCW) with a conversion yield of 0.034 g TAPS/(g glycerol) within 48 h (Figure 5A), whereas the wild-type produced 0.8 g/L of TAPS (29.9 mg-TAPS/g-DCW) with a conversion yield of 0.023 g TAPS/(g glycerol) within 48 h (Figure 5B). Mutant 736 and the wild-type strain reached an OD of ∼48 with μ_max_ = 0.36/h and 0.40/h, respectively, after glycerol was completely consumed. In batch fermentation with 130 g/L of glycerol, mutant 736 produced 9.1 g/L of TAPS (218.7 mg-TAPS/g-DCW) with a conversion yield of 0.08 g of TAPS/(g glycerol) within 120 h (Figure 5C), whereas the wild-type produced 1.7 g/L of TAPS (52.2 mg-TAPS/g-DCW) with a conversion yield of 0.02 g TAPS/(g glycerol) in 120 h (Figure 5D). The high initial glycerol concentration (130 g/L) reduced the μ_max_ of the wild-type strain (0.17/h) by 53% and that of mutant 736 (0.28/h) by 30%, when compared with the μ_max_ for fermentation at the initial glycerol concentration of 35 g/L. For the highest TAPS production, fed-batch bioreactor fermentation of mutant 736 was performed in the YMgISC medium using the glycerol-feeding method. Mutant 736 grew to an OD_600_ of ∼142 with μ_max_ = 0.36/h (Figure 6A). The mutant 736 produced 17.7 g/L of TAPS (259.6 mg-TAPS/g-DCW) during fed-batch fermentation for 240 h (Figure 6B).

## Discussion

As one of the strategies for increasing TAPS production in *W. ciferrii*, we exposed the cells to γ-rays to obtain *W. ciferrii* mutants producing more TAPS. Among the suitable mutants, a mutant named 736 was selected and its TAPS production and physiological properties were compared with those of the wild-type strain.

Mutant 736 produced up to 9.1 g/L of TAPS (218.7 mg-TAPS/g-DCW) during bioreactor fermentation at 130 g/L of glycerol with a higher conversion yield and faster growth rate when compared with the wild-type strain (1.7 g/L; 52.2 mg-TAPS/g-DCW). Higher TAPS production in mutant 736 than in the wild-type was indirectly proved by comparing appearances of the colonies of mutant 736 and wild-type yeasts grown on agar plates (Figure 2A). Fed-batch bioreactor fermentation by mutant 736 yielded even higher production of TAPS (17.7 g/L; 259.6 mg-TAPS/g-DCW), which is the highest titer reported in *W. ciferrii* (Schorsch et al., 2012) and in a recombinant *Y. lipolytica* (Han et al., 2020). As the culture medium and conditions were not fully optimized for the bioreactor fermentation by mutant 736, further systematic optimization of media composition and culture conditions, including temperature and pH, may enhance the current productivity. Furthermore, utilizing crude glycerol (or biodiesel derived waste glycerol) as a carbon source can reduce the commercial scale production cost of TAPS from *W. ciferrii*, as seen in yeast fermentations for lipid production from glycerol (Chatzifragkou et al., 2011). In comparison with typical oleaginous yeasts, *W. ciferrii* wild-type and mutant 736 showed relatively higher biomass yield on glycerol (Y_x/__gly_, g/g) (Table 2), which suggests the high feasibility of the cost-effective commercial scale production of TAPS from glycerol. These optimization strategies are promising because TAPS production in mutant 736 was significantly enhanced by the addition of serine and CaCl_2_ into culture media, as was the case for the wild-type. The positive effect of serine can be explained as follows: serine, one of the two precursors, may be utilized to a greater extent in TAPS biosynthesis (Figure 1). It should be mentioned that enhanced TAPS biosynthesis should be balanced by the flux of two precursors: serine and palmitoyl-CoA. Notably, C16:0 fatty acid was found to be less abundant in mutant 736 than in the wild type (Figure 3). As in TAPS biosynthesis, an activated form of the C16:0 fatty acid is the other precursor (palmitoyl-CoA), greater flux of the palmitoyl-CoA pool into TAPS biosynthesis than that in C16:0 fatty acid biosynthesis may enhance TAPS production and decrease cellular C16:0 fatty acid formation. Of note, mutant 736 seemed to increase the production of C18:1 and C18:2 fatty acids to compensate for the lower abundance of the C16:0 fatty acid and for maintaining membrane function and fluidity. Although a significant change in the cellular fatty-acid profile was observed here, the morphology of wild-type and mutant 736 cells was similar, as evident in our scanning electron micrographs.

**TABLE 2:** Comparison of growth parameters of selected yeasts grown on glycerol.

**Strain**	**Time (h)**	**Gly_cons_ (g/L)**	**X (g/L)**	**Y_X/gly_ (g/g)**	**Growth mode**	**References**
*W. ciferrii*	120	63.3	33.9	0.5	Batch	This study
*W. ciferrii* mut736	120	100.6	41.4	0.4	Batch	This study
*W. ciferrii* mut736	240	286.6	68.2	0.2	Fed-batch	This study
*Rhodosporidium toruloides*	264	100.1	31.3	0.3	Flask	Papanikolaou et al., 2017a
*Rhodotorula* sp. LFMB22	187	26.0	8.1	0.3	Flask	Chatzifragkou et al., 2011
*Yarrowia lipolytica* LFMB19	120	28.2	7.9	0.3	Flask	Chatzifragkou et al., 2011
*Yarrowia lipolytica*	48	80.0	42.2	0.5	Fed-batch	Fontanille et al., 2012
*Pichia membranifaciens*	24	2.8	1.6	0.6	Flask	Chatzifragkou et al., 2011
*Pichia membranifaciens*	138	24.0	7.8	0.3	Flask	Chatzifragkou et al., 2011
*Thamnidium elegans*	450	43.7	14.3	0.3	Flask	Chatzifragkou et al., 2011
*Thamnidium elegans*	550	72.6	16.3	0.2	Flask	Chatzifragkou et al., 2011
*Yarrowia deformans*	144	100.0	42.0	0.4	Flask	Rakicka et al., 2016
*Cryptococcus curvatus*	288	134.3	50.0	0.6	Fed-batch	Liang et al., 2010

*Gly_*cons*,_ glycerol consumed (g/L); X, dried cell biomass (g/L); Time, fermentation period (h); Y_*X/gly*_, yield of dried cell biomass per glycerol consumed (g/g).*

In contrast to the effects of serine, the positive effect of calcium ions is not fully understood. Calcium ions have a variety of cellular roles, including those associated with cell proliferation, signaling, programmed cell death, and gene expression in eukaryotic cells (Lotan et al., 1976; Saltukoglu and Slaughter, 1983; Cui et al., 2009). Consequently, the intracellular accumulation of calcium ions may be indirectly associated with TAPS biosynthesis at some points in the pathway.

Finally, we found that there are no mutations in eight TAPS biosynthesis-related genes (that is, there is no alteration of the inherent activity or regulatory properties of the enzymes) in mutant 736; however, among the eight genes, we detected 30% higher mRNA expression of *TSC10* in mutant 736 than in the wild type. This finding indicates that the enhancement of TAPS biosynthesis in mutant 736 might be a result of systems level genetic and physiological alterations of a complicated metabolic network. Therefore, systems level studies, including whole-genome resequencing and high-throughput RNA sequencing, are needed and are now underway. Reverse metabolic engineering based on the obtained genetic and physiological variation information can make mutant 736 a more suitable strain for commercial TAPS production.

## Data Availability Statement

The raw data supporting the conclusions of this article will be made available by the authors, without undue reservation.

## Author Contributions

JC, J-IC, and PL conceptualized the study. JC and HH were in charge of the methodology, validation, and formal analysis. HH and PL prepared and wrote the original draft. JC and PL reviewed, edited, and wrote the manuscript. JC handled the visualization. PL supervised the study and was in charge of the funding acquisition. All authors have read and agreed to the published version of the manuscript.

## Conflict of Interest

The authors declare that the research was conducted in the absence of any commercial or financial relationships that could be construed as a potential conflict of interest.

## Footnotes

1 https://www.ncbi.nlm.nih.gov/nuccore/CAIF01000001/CAIF01000364
