# Supplementary material for: Differences in the Fatty Acid Profile, Morphology, and Tetraacetylphytosphingosine-Forming Capability Between Wild-Type and Mutant Wickerhamomyces ciferrii
Source: Front Bioeng Biotechnol. 2021 Jun 9;9:662979. doi: 10.3389/fbioe.2021.662979 (PMC8220092; doi:10.3389/fbioe.2021.662979)
Supplement: Supplementary file 1 [file Data_Sheet_1.DOCX]

Supplementary Material

## Supplementary Figures

**
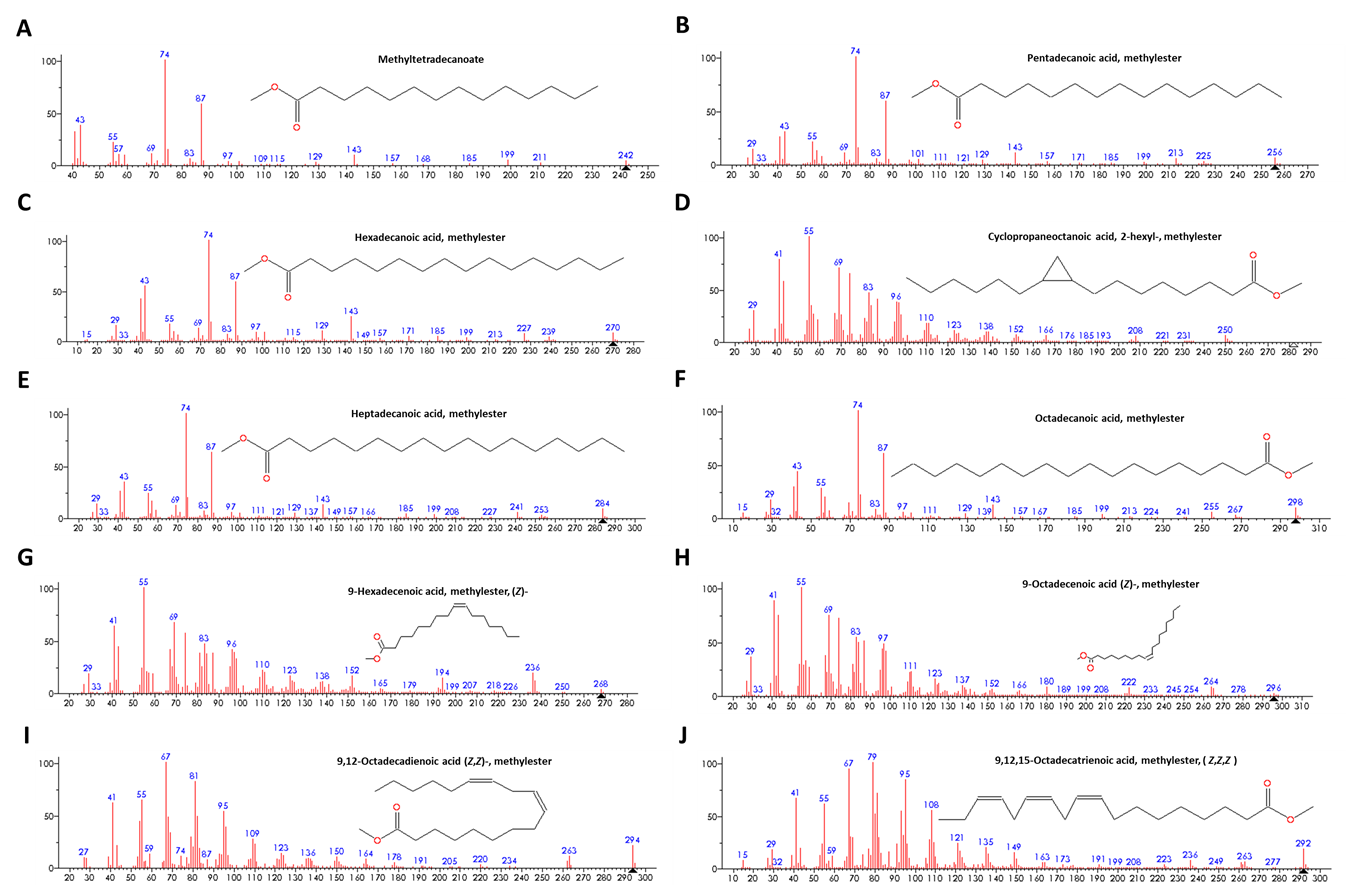
**

**Supplementary Figure 1 |** Mass fragmentation patterns of total fatty acids from *Wickerhamomyces ciferrii*, the wild-type strain and mutant 736. Each fatty acid that was separated with GC was automatically annotated using the NIST (National Institute of Standards and Technology) library software.


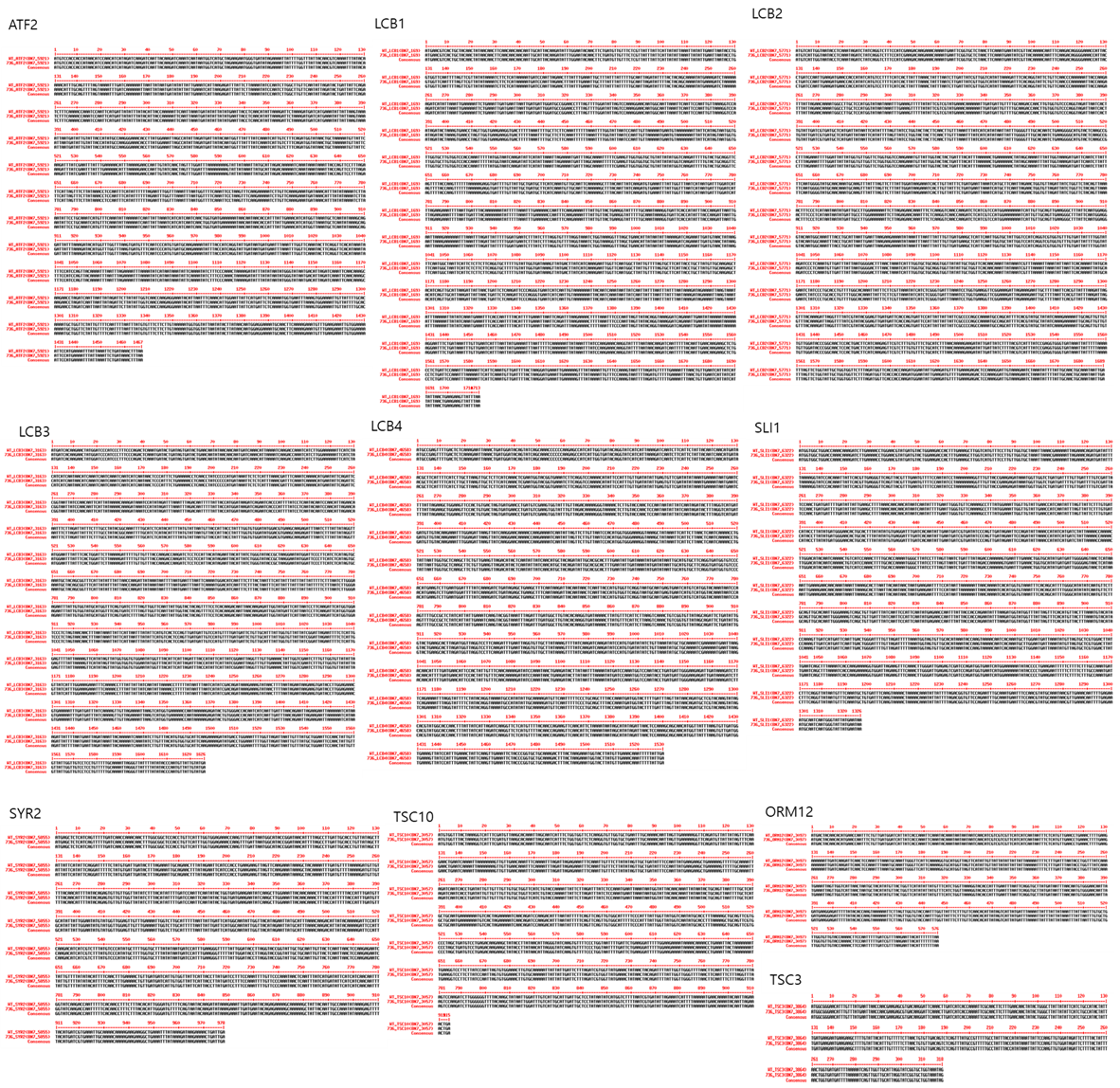


**Supplementary Figure 2 |** Amino acid sequences of 10 proteins from *W. ciferrii*, the wild type and mutant 736, were aligned in the ClustalW software after Sanger sequencing of the 10 corresponding genes in the wild type and mutant 736.

## Supplementary tables

**Supplementary Table 1 |** Mass fragmentation patterns of total fatty acids from *W. ciferrii*, the wild type and mutant 736. Each fatty acid separated with GC was automatically annotated in the NIST library software.

| **Fatty acid,** | **Common name** |  | **Wild-type** | | **Mutant 736** | | ***t*** | ***p* value** | **CV (%)** | |
| --- | --- | --- | --- | --- | --- | --- | --- | --- | --- | --- |
|  |  |  | **Mean** | **SD** | **Mean** | **SD** |  |  | **Wild-type** | **Mutant 736** |
| **Saturated fatty acids** | | | | | | | | | | |
| Methyl tetradecanoate | C14:0 methyl ester |  | 0.77 | 0.06 | 0.51 | 0.05 | 6.70 | <0.001 | 7.89 | 9.38 |
| Pentadecanoic acid, methyl ester | C15:0 methyl ester |  | 0.37 | 0.03 | 0.18 | 0.05 | 7.63 | <0.001 | 6.87 | 30.63 |
| Hexadecanoic acid, methyl ester | C16:0 methyl ester |  | 23.0 | 0.50 | 19.5 | 0.39 | 33.9 | <0.0001 | 2.18 | 2.00 |
| Cyclopropaneoctanoic acid, 2-hexyl,  methyl ester | 9,10-cpa-C17:0 methyl ester |  | 0.90 | 0.05 | 1.31 | 0.24 | 4.06 | <0.01 | 5.03 | 18.00 |
| Heptadecanoic acid, methyl ester | C17:0 methyl ester |  | 0.19 | 0.02 | 0.29 | 0.10 | 2.55 | <0.05 | 9.61 | 32.76 |
| Octadecanoic acid, methyl ester | C18:0 methyl ester |  | 3.40 | 0.15 | 2.44 | 0.15 | 10.6 | <0.0001 | 4.43 | 6.29 |
| **Monounsaturated fatty acids** | | | | | | | | | | |
| 9-Hexadecenoic acid(*Z*), methyl ester | C16:1 methyl ester |  | 5.33 | 0.10 | 4.55 | 0.14 | 10.5 | <0.0001 | 1.88 | 3.02 |
| 9-Octadecenoic acid(Z), methyl ester | C18:1 methyl ester |  | 37.4 | 1.26 | 39.9 | 2.82 | 2.01 | <0.1 | 3.37 | 7.07 |
| **Polyunsaturated fatty acids** | | | | | | | | | | |
| 9,12-Octadecadienoic acid (Z,Z), methyl ester | C18:2-delta-9Z,12Z methyl ester |  | 23.7 | 0.45 | 26.2 | 0.39 | 12.3 | <0.0001 | 1.88 | 1.48 |
| 9,12,15-Octadecatrienoic acid (Z,Z,Z), methyl ester | C18:3-9Z,12Z,15Z methyl ester |  | 1.23 | 0.10 | 1.40 | 0.15 | 2.72 | <0.05 | 8.46 | 10.88 |
